# The Role of Non-Coding RNAs in Controlling Cell Cycle Related Proteins in Cancer Cells

**DOI:** 10.3389/fonc.2020.608975

**Published:** 2020-11-30

**Authors:** Soudeh Ghafouri-Fard, Hamed Shoorei, Farhad Tondro Anamag, Mohammad Taheri

**Affiliations:** ^1^ Department of Medical Genetics, Shahid Beheshti University of Medical Sciences, Tehran, Iran; ^2^ Department of Anatomical Sciences, Faculty of Medicine, Birjand University of Medical Sciences, Birjand, Iran; ^3^ Faculty of Medicine, Tabriz University of Medical Sciences, Tabriz, Iran; ^4^ Urology and Nephrology Research Center, Shahid Beheshti University of Medical Sciences, Tehran, Iran; ^5^ Urogenital Stem Cell Research Center, Shahid Beheshti University of Medical Sciences, Tehran, Iran

**Keywords:** cell cycle, microRNA, long non coding RNA, expression, polymorphism

## Abstract

Cell cycle is regulated by a number of proteins namely cyclin-dependent kinases (CDKs) and their associated cyclins which bind with and activate CDKs in a phase specific manner. Additionally, several transcription factors (TFs) such as E2F and p53 and numerous signaling pathways regulate cell cycle progression. Recent studies have accentuated the role of long non-coding RNAs (lncRNAs) and microRNAs (miRNAs) in the regulation of cell cycle. Both lncRNAs and miRNAs interact with TFs participating in the regulation of cell cycle transition. Dysregulation of cell cycle regulatory miRNAs and lncRNAs results in human disorders particularly cancers. Understanding the role of lncRNAs, miRNAs, and TFs in the regulation of cell cycle would pave the way for design of anticancer therapies which intervene with the cell cycle progression. In the current review, we describe the role of lncRNAs and miRNAs in the regulation of cell cycle and their association with human malignancies.

## Introduction

Cell division has a fundamental role in the development multicellular organisms. This process is accomplished through orderly sequences of happenings that together with each other make the “cell cycle”. This cycle comprises precise duplication of the genome throughout the DNA synthesis stage (S phase), and separation of whole sets of chromosomes to one of the daughter cells in the mitosis stage (M phase). Two “Gap” phases also exist in the cell cycle. The first one (G_1_) links the accomplishment of the M phase to the commencement of S phase in the succeeding cycle. G_2_ splits the S and M stages. Cells residing in the G_1_ phase might momentarily or enduringly exit the cell cycle and go in an inactive or blocked phase namely G_0_ (1). In the mammalian cells, cell cycle is regulated by a number of proteins namely cyclin-dependent kinases (CDKs) and their associated cyclins which bind with and activate CDKs in a phase specific manner (2). Cyclins A and E are activating factors for CDK2. Cyclin B binds with CDK1. Finally, CDK4/6 is activated by cyclin Ds. Binding of cyclins with CDKs leads to phosphorylation of CDKs target proteins which finally permits progression through cell cycle (2). In addition to cyclins, Wee1 kinase and CDC25 phosphatase regulate activity of CDKs by phosphorylation and dephosphorylation reactions, respectively (3). Activity of CDKs and cell cycle progression are inhibited by a number of factors such as p15^ink4b^, p16^ink4a^, p18^ink4d^, p21^Cip1^, p27^Kip1^, and p57^Kip2^. These factors have a specific binding affinity for cyclin-CDK complexes (4–6). Progression through each phase of cell cycle is regulated by CDKs and their associated proteins/pathways. For instance, MAPK pathway induced by growth factors enhances transcription of cyclin Ds in the G1 phase, leading to activation of CDK4/6 (7). The protein complex formed by cyclin Ds and CDK4/6 phosphorylates retinoblastoma protein (pRB), p107 and p130, in the final stages of G1 phase, thus releasing E2F and enhancing E2F-dependent expression of growth-stimulating genes (7). At the G1/S boundary, the complex constructed by cyclin E and CDK2 phosphorylates pRB and other proteins participating in the regulation of DNA replication to facilitate G1/S transition (8). Cyclin B-CDK1 complex has several functions such as accomplishment of the G2 phase processes (9), negative regulation of cytokinesis (10), and coordination of mitotic-related procedures in the nucleus and the cytoplasm (11). This complex has a number of targets such as the anaphase-promoting complex/cyclosome (APC/C) (10). In addition to CDKs, cyclins, and the APC/C which directly regulate cell cycle progression, other molecules are involved in this process. For instance, p53 functions in numerous stages to warrant that cells do not bring their abnormal DNA through cell division. It halts the cell cycle at the G1 checkpoint through stimulating synthesis of CDK inhibitor (CKI) proteins. These proteins attach CDK-cyclin complexes and inhibit their function extending the time for the activation of DNA repair system. It also induces DNA repair enzymes. If DNA damage cannot be fixed, p53 induces cell apoptosis to prevent transmission of the damaged DNA to the daughter cells (12).

Several lines of evidence point to the role of non-coding RNAs (ncRNAs) in the regulation of expression or activity of the above-mentioned proteins (13). Cyclins, CDKs and their inhibitors, are targets of regulation by ncRNAs at different levels including transcriptional and post-transcriptional levels (13). Being classified mainly based on their sizes, ncRNAs include micro RNAs (miRNAs) and long non-coding RNAs (lncRNAs). LncRNAs are longer than 200 nucleotides. According to their structural features, lncRNAs are categorized into different classes among them are intergenic, intronic and natural antisense lncRNAs (14). In addition to their regulatory roles on gene expression at transcriptional and post-transcriptional levels, lncRNAs act as protein scaffolds to regulate interactions between proteins (15). Expressions of a number of lncRNAs are stimulated by DNA damage. These transcripts contribute in DNA damage responses and carcinogenic processes (13). Meanwhile, there is a reciprocal interaction between miRNAs and a number of cell cycle regulators in a way that miRNAs regulate expression of cell cycle regulators, and expression of miRNA is regulated by cell-cycle-dependent transcription factors (16). Such interactive network is implicated in the pathogenesis of a number of disorders particularly cancer. Most of miRNAs are first transcribed from their encoding genes into primary miRNAs. Then, they are changed to precursor miRNAs, and eventually mature miRNAs. These steps are performed in both nucleus and cytoplasm. Mechanistically, miRNAs interact with the 3′ UTR of their target transcripts resulting in degradation their or inhibition of their translation. Yet, miRNAs binding with the 5′ UTR, coding regions, and promoters, has also been demonstrated (17). In the current review, we describe the role of lncRNAs and miRNAs in the regulation of cell cycle and their association with human malignancies.

## LncRNAs and Cell Cycle Control

Numerous lncRNAs have been shown to regulate cell cycle progression directly through modulation of expression of CDKs/cyclins or indirectly through regulation of TFs that control expression of CDKs/cyclins. For instance, the known oncogenic lncRNA MALAT1 regulates cell cycle progression at G1 phase since its knock down has resulted in cell cycle arrest at this step and enhanced expression of cell cycle inhibitors p53, p16, p21, and p27, while suppressing expression of cyclin A2 and CDC25A (18). Moreover, this lncRNA has a crucial role in up-regulation of expression of B-Myb, an oncogenic TF participating in G2/M progression (18). Thus, MALAT1 regulates cell cycle progression in different phases through modulation of expression of cell cycle regulators.

ANRIL is an lncRNA being transcribed from the INK4 locus in an antisense direction to p15 (19). This lncRNA participates in epigenetic suppression of expression of the INK4 locus through recruitment of polycomb repression complex 2 (PRC2). Such function specifically represses expression of p15 (20). Expression of CDK inhibitors is also regulated by other lncRNAs including lncRNA-HEIH. This up-regulated lncRNA in the hepatitis B virus-associated hepatocellular carcinoma decreases expression of p15, p16, p21, and p57, through cooperation with EZH2, thus regulating cell cycle transition at G0/G1 (21).

HOTAIR has been shown to regulate expression of genes which are principally associated with cell cycle progression (22). HOTAIR silencing has led to cell cycle arrest at G0/G1 in addition to modulation of expression of cell cycle-related proteins (22). Experiment in esophageal squamous cell carcinoma cells has also verified the impact of HOTAIR silencing on suppression of cell proliferation and induction of G1 cell cycle arrest. This lncRNA serves as a molecular sponge to suppress miR-1 expression and subsequently increase expression of cyclin D1 (23). In ovarian cancer cells, HOTAIR increases expression of cyclin D1 and cyclin D2 through negatively regulating miR-206 expression (24).

A number of lncRNAs have been shown to regulate expression of p53 thus affecting cell cycle regulation by this TF. These lncRNAs include both oncogenic and tumor suppressor ones. Examples from the former group include PVT1 and ANRIL which enhance MDM2-associated degradation of p53 (25, 26). On the other hand, LOC572558 enhances expression of p53 through regulation of its phosphorylation (27). Moreover, MEG3 RNA interacts with the promoter region of p21 to increase p53 accumulation (28). Meanwhile, p53 as a TF can alter expression of several lncRNAs. For example, the lncRNA-p21 has been shown to be transcribed from a genomic region adjacent to p21^Cip1^. This lncRNA is a direct target of p53. LncRNA-p21 silencing has affected expression of a number of p53-target genes with the exception of p21 gene (29). Moreover, the lncRNA PANDA is transcribed from a promoter region of p21 in response to DNA damage through a p53-dependent route (30). Therefore, lncRNAs exert functional roles both at upstream and downstream of cell cycle-associated TFs such as p53.

The lncRNA EMS has been identified as a direct target of c-Myc. This lncRNA acts as an oncogenic transcript that facilitates G1/S transition. Functional studies have shown interaction between EMS and the RNA binding protein RALY to increase the stability of E2F1 transcript and enhance its expression (31). The oncogenic lncRNA MIR31HG has been shown to promote cell proliferation, facilitate cell cycle progression, and suppress cell apoptosis. This lncRNA modulates cell cycle transition through regulation of HIF1A and p21 expressions (32). In addition, SHNG3 is another dysregulated lncRNA in diverse cancers. This lncRNA has higher expression in glioma tissues and cell lines compared with normal counterparts. Forced over-expression of SNHG3 has increased proliferation, quickened cell cycle progression, and suppressed cell apoptosis *via* silencing KLF2 and p21 through recruitment of EZH2 to their promoter (33). FOXD2-AS1 is another oncogenic lncRNA whose knock down results in cell cycle arrest in the G0/G1 stage, inhibition of colony development, cell proliferation, and suppression of tumor growth in the xenograft model. FOXD2-AS1 and reduced expression of CDKN1B through recruitment of EZH2 to its promoter region (34). ROR1-AS1 in an up-regulated lncRNA in colon cancer tissues, particularly in stage III and IV and more massive tumors. Forced over-expression of ROR1-AS1 has increased cell proliferation, reduced the G0/G1 phase time of cell cycle, and inhibited apoptosis. This lncRNA can bind to EZH2 and suppress expression of DUSP5 (35).

**Figure 1:**
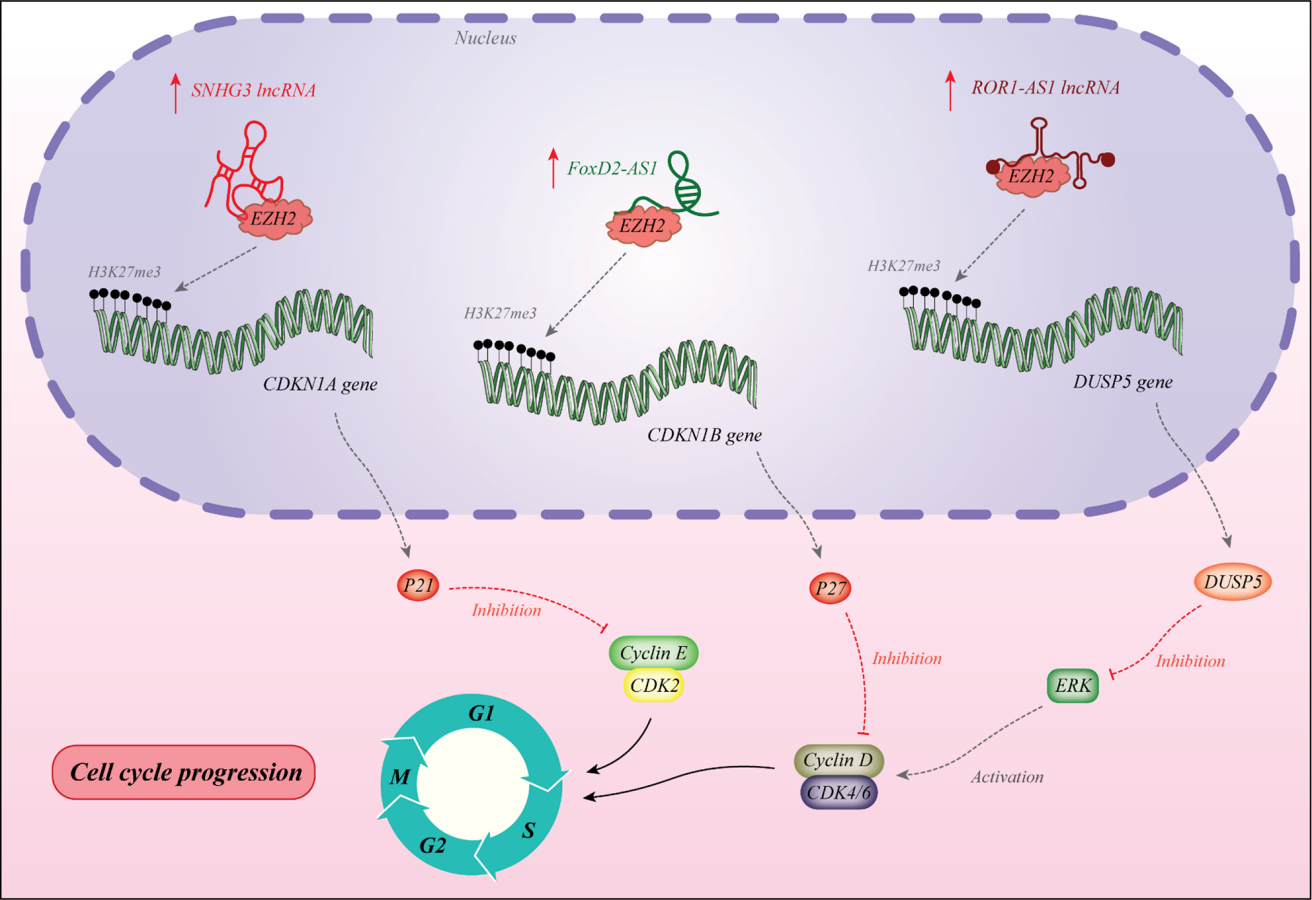
The lncRNA SNHG3 recruits EZH2 to the promoter of CDKN1A to induce H3K27me3 and decrease expression of this gene. This gene encodes the p21 protein which is an inhibitor of cyclin E/CDK2 (33). The lncRNA FOXD2-AS1 enhances recruitment of EZH2 to the promoter of CDKN1B and decreases its expression *via* H3K27me3. Therefore, it down-regulates p27 which is an inhibitor of cyclin D (34). These two lncRNAs promote cell progression at G1/S point. Over-expression of ROR1-AS1 has increased cell proliferation, reduced the G0/G1 phase time of cell cycle, and inhibited apoptosis. This lncRNA can bind to EZH2 and suppress expression of DUSP5 (35).

**Figure 1** shows the molecular mechanism of involvement of a number of lncRNAs in cell cycle regulation. These lncRNAs recruit EZH2 to the promoter regions of their target genes.

**Table 1** shows the results of studies which assessed the role of lncRNAs in cell cycle control.

**Table 1:** The role of lncRNAs in cell cycle control (ANTs, Adjacent normal tissues).

Cancer Type	lncRNA	Species/number of samples	Targets/Regulators	Involved pathways	Function	Ref
Prostate Cancer (PCa)	LOXL1-AS1	–	CCND1, miR-541-3p	–	LOXL1-AS1 *via* targeting miR-541-3p and CCND1 could regulate prostate cancer cell proliferation and cell cycle progression.	(36)
Pca	NR2F2-AS1	60 pairs of Pca and ANTs	CDK4	–	NR2F2-AS1 by regulating CDK4 could promote cancer cell proliferation in Pca.	(37)
Pca	SNHG1	30 pairs of Pca and ANTs	miR-199a-3p, CDK7,	–	SNHG1 *via* regulating miR-199a-3p/CDK7 axis could promote cell proliferation in prostate cancer.	(38)
Pca	MALAT1	–	CDK6, Cyclin D1, p27, AR, miR-320b	–	Knockdown of MALAT1 *via* androgen receptor signaling could inhibit cell cycle progression in Pca cells.	(39)
Esophageal Squamous Cell Carcinoma (ESCC)	HOTAIR	32 pairs of ESCC and ANTs	miR-1, CCND1, Ago2	–	Knockdown of HOTAIR *via* targeting miR-1/CCND1 axis could repress proliferation and lead to G1 cell cycle arrest in ESCC cells.	(23)
Hepatocellular Carcinoma (HCC)	PCAT6	TCGA database	DCAF13, SNRPB2, RPS8, FKBP1A, PCNA, CCND1, BCL-2	Wnt, HIF-1	Upregulation of PCAT6 could decrease the percentage of cells in the G2/M phase. Hence, PCAT6 *via* the regulation of cell cycle arrest and apoptosis could promote proliferation in HCC.	(40)
HCC	FOXD2-AS1	105 pairs of HCC and ANTs	CDK2, Cyclin E1, CDK4, Cyclin D1, CDKN1B, EZH2	–	Knockdown of FOXD2-AS1 *via* targeting CDKN1B/EZH2 could arrest the cell cycle in the G0/G1 phase.	(34)
HCC	HOXD-AS1	Mouse/human; 20 pairs of HCC and ANTs	Cyclin A1, Cyclin B1, Cyclin D1, BCL-2, BAX, MMP1/2/9	MEK/ERK	Knockdown of HOXD‐AS1 could induce S or/and G2/M phase cell cycle arrest, and also could suppress the proliferation, migration, and invasion.	(35)
Renal Cell Carcinoma (RCC)	CRNDE	15 pairs of RCC and ANTs	APC2, AXIN2, WNT2B, WNT4, SNAIL2, FZD4, CRNDE, CCND1, CCNE1	Wnt/β-catenin	CRNDE by regulating the cell cycle the transition from the G0/G1 stage to the S stage *via* Wnt/β-catenin signaling could promote cell proliferation.	(41)
Adrenocortical carcinoma (ACC)	HOTAIR	77 ACC tissues and 30 normal adrenal tissues, GEO database	p-GSK3β, p-Rb, Cyclin D1	–	HOTAIR *via* regulating cell cycle could be involved in the development and progression of ACC.	(42)
Melanoma	UCA1	Melanoma patients (n=18) normal tissues (n=20),	miR-507, FOXM1	–	UCA1 *via* miR-507-FOXM1 axis could be involved in cell proliferation, invasion, and G0/G1 cell cycle arrest in melanoma.	(43)
Melanoma	GAS5	47 pairs of melanoma and ANTs	Cyclin D1, CDK4, p27, Bcl-2, p21, Caspase-3, G6PD	–	Knockdown of GAS5 by inducing G1/S cell cycle progression could increase melanoma cell proliferation.	(31)
Head & Neck Squamous Cell Carcinoma (HNSCC)	MIR31HG	–	HIF1A, p21, p53, p27, CCND1	–	MIR31HG by targeting HIF1A/P21 axis could facilitate HNSCC cell proliferation and tumorigenesis.	(32)
Glioma	HOXD-AS2	Mouse	c-Myc, Cyclin D1, Cyclin A, E2F1, p27	–	Knockdown of HOXD‐AS2 by inducing cell‐cycle G1 arrest could inhibit glioma cell growth.	(44)
Glioma	ANRIL	30 pairs of glioma and ANTs	Caspase-3/8/9,miR-203a, CDK2, Bcl-2, p21, c-Myc	AkT	Knockdown of ANRIL *via* targeting miR-203a could induce anoikis and cell cycle arrest in the G0/G1 phase.	(45)
Glioma	SNHG3	60 pairs of glioma and ANTs	KLF2, p21	–	SNHG3 by silencing KLF2 and p21 could enhance the malignant progress of glioma. Knockdown of SNHG3 could induce cell cycle arrest in the G0/G1 phase.	(33)
Ameloblastoma (AB)	ENST00000512916	26 pairs of AB and ANTs	HOXC13, Cyclin D1, CDK2/4/6, Tubulin	–	lncRNA ENST00000512916 could promote cell proliferation, migration, and cell cycle progression of AB.	(46)
Osteosarcoma (OS)	FLVCR-AS1	48 pairs of OS and ANTs	CCND1, miR-381-3p	–	FLVCR-AS1 by targeting miR-381-3p/CCND1 could promote osteosarcoma growth.	(47)
Osteosarcoma (OS)	LINC01296	30 pairs of OS and ANTs	Cyclin D1, Cyclin E1, CDK2/4	–	LINC01296 *via* targeting cyclin D1 could regulate the proliferation, metastasis, and cell cycle of osteosarcoma.	(48)
Breast Cancer	LINC00668	TCGA dataset	CDK4, Bcl-2, p21	AKT/mTOR	LINC00668 by inhibiting apoptosis and accelerating cell cycle could promote the progression of breast cancer.	(49)
Breast Cancer	MALAT1	Mouse/human; 40 pairs of breast cancer and ANTs	miR-124,CDK4, E2F1	–	MALAT1 *via* miR-124/CDK4/E2F1 axis could enhance breast cancer progression.	(50)
Breast Cancer	RUSC1-AS1	48 pairs of breast cancer and ANTs	KLF2, CDKN1A	–	Knockdown of RUSC1-AS1 *via* inhibiting cell cycle progression through the KLF2/CDKN1A axis could induce apoptosis in breast cancer cells.	(51)
Breast Cancer	SNHG6	45 pairs of breast cancer and ANTs	Cyclin D1, PCNA, Snail, Vimentin, E-cadherin	–	Overexpression of SNHG6 could promote cell cycle progression, proliferation, migration, and EMT of breast cancer cells.	(52)
Pancreatic cancer (PaC)	TUG1	Mouse/human; 42 pairs of PaC and ANTs	EZH2, MT2A, RND3	–	Knockdown of TUG1 by targeting the RND3/MT2A axis could block the cell cycle and accelerated apoptosis in PaC cells.	(53)
Oral Squamous Cell Carcinoma (OSCC)	NEAT1	30 pairs of OSCC and ANTs	miR-365, RGS20, cyclin D1, Vimentin, E-cadherin, N-cadherin,	–	Knockdown of NEAT1 by regulating miR-365/RGS20 axis could induce cell cycle arrest at the G0/G1 phase and inhibit cell proliferation and invasion.	(54)
Colorectal cancer (CRC)	CLMAT3	–	Cdh1, p27Kip1, Skp2	–	Knockdown of CLMAT3 could induce the G0/G1 cell-cycle arrest in CRC cells.	(55)
CRC	XIAP-AS1	75 pairs of CRC and ANTs	E-cadherin, ZO-1, vimentin, N-cadherin, p63, Cyclin D1, Cyclin E, c-Myc, Cyclin A	–	Knockdown of XIAP-AS1 could arrest the cell cycle at the G0/G1 phase, and be involved in cell proliferation and invasion in CRC.	(53)
CRC	NR2F2-AS1	60 pairs of CRC and ANTs	Cyclin D1	–	Knockdown of NR2F2-AS1 *via* downregulating Cyclin D1 could induce cell cycle arrest in the G0/G1 phase in CRC.	(56)
CRC	NR2F2-AS1	63 pairs of CRC and ANTs	CDK6	–	Knockdown of NR2F2−AS1 could induce G1 arrest by downregulating CDK6 in CRC.	(57)
CRC	ROR1-AS1	75 pairs of CRC and ANTs	DUSP5, CDKN1A, EZH2	–	After overexpression of ROR1-AS1, the G0/G1 phase time of cell cycle was shortened. Hence, ROR1-AS1 by suppressing the DUSP5/CDKN1A axis could promote CRC cell proliferation.	(35)
CRC	SNHG1	86 pairs of CRC and ANTs	Bax, p53, p21	–	SNHG1 by affecting P53 could promote cell proliferation in CRC. Knockdown of SNHG1 could induce G0/G1 phase arrest.	(58)
CRC	STEAP3-AS1	Mouse/TCGA database	CDKN1C, STEAP3, CDK2, CDK4, Cyclin E2, acetyl-H3	–	STEAP3-AS1 by affecting CDKN1C could modulate cell cycle progression in CRC. Knockdown of STEAP3-AS1 could arrest CRC cells at the G0–G1 phase.	(59)
Papillary Thyroid Carcinoma (PTC)	SNHG12	Mouse/human; 30 pairs of PTC and ANTs	MMp2, Cyclin D1	Wnt/β-catenin	Knockdown of SNHG12 *via* regulating Wnt/β-catenin signaling could block cell cycle progression at the G1-G0 phase in PTC.	(60)
Thyroid Carcinoma (TC)	PANDAR	75 pairs of TC and ANTs	Chk1, Cdc25A, Cyclin D1, Bax, Bcl-2	–	Knockdown of PANDAR could promote apoptosis and suppress the proliferation and cell cycle in TC cells.	(61)
Gastric Cancer (GC)	HNF1A-AS1	GC tissues (n=99) nontumorous gastric tissues (n=8),	EGR1, miR-661, CDC34, CDK2/4, Cyclin E1, p21	–	HNF1A-AS1 through modulation of the cell cycle could contribute to gastric cancer progression.	(62)
GC	CASC11	80 pairs of GC and ANTs	miR-340-5p, CDK1, Cyclin A2, Cyclin B1, PIK1	–	CASC11 by regulating cell cycle pathway *via* the miR-340-5p/CDK1 axis could promote GC cell proliferation, migration, and invasion.	(63)
Non-Small Cell Lung Cancer (NSCLC)	HNF1A-AS1	Mouse/human; 60 pairs of NSCLC and ANTs	miR-149-5p, Cdk6, p21, p27, Cyclin D1	–	HNF1A‐AS1/miR‐149‐5p/Cdk6 axis could participate in NSCLC progression.	(64)
Endometrial Cancer	NEAT1	GEO database	c-Myc, MMP9, LEF1 miR-146b-5p	Wnt/β-catenin	Progesterone by regulating the NEAT1/miR-146b-5p axis *via* Wnt/β-catenin pathway could inhibit endometrial cancer cell cycle and viability.	(65)
Acute Myeloid Leukemia (AML)	HOTTIP	Bone marrow blood samples from 80 AML patients and 24 healthy controls	miR-608, DDA1	–	HOTTIP *via* regulating cell cycle could promote the proliferation of AML cells.	(65)
Larynx Squamous Cell Carcinoma (LSCC)	CDKN2B-AS1	60 pairs of LSCC and ANTs	miR-324-5p, PARP, Caspase-3, p21, ROCK1, PCNA	–	Overexpression of CDKN2B-AS1 *via* the miR-324-5p/ROCK1 axis by regulating cell cycle could promote LSCC cell proliferation.	(66)

The interaction between lncRNAs and cell cycle-related proteins can alter response of cancer cells to chemotherapeutic agents. For instance TUG1 has a role in induction of chemoresistance in small cell lung cancer cells through regulation of LIMK2b expression. Knockdown of TUG1 has resulted in the accumulation of cells at G1-phase (67). NNT-AS1 *via* MAPK/Slug pathway could be involved in cisplatin chemoresistance in non-small cell lung cancer (68). **Table 2** summarizes the results of studies which assessed the role of lncRNAs in this regard.

**Table 2:** Role of cell-cycle related lncRNAs in chemoresistance (ANTs, Adjacent normal tissues).

Cancer Type	lncRNA	Human/Animal	Targets/Regulators	Involved pathways	Function	Ref
Small Cell Lung Cancer (SCLC)	TUG1	33 primary cancerous and 11 ANTs	LIMK2b, EZH2	–	Knockdown of TUG1 led to a significant accumulation of cells at G1-phase. TUG1 by regulating LIMK2b *via* EZH2 could be involved in cell growth and chemoresistance of SCLC.	(67)
Non-Small Cell Lung Cancer (NSCLC)	NNT-AS1	10 pairs of drug-resistant and non-resistant tissues	–	MAPK/Slug	NNT-AS1 *via* MAPK/Slug pathway could be involved in cisplatin chemoresistance in NSCLC. Interfering in the expression of NNT-AS1 could promote the cell cycle arrest of drug-resistant cells.	(68)
Cervical Cancer (CC)	NEAT1	Mouse/human; 72 pairs of CC and ANTs	miR-193b-3p, p21,Cyclin D1, Caspase-3	–	Knockdown of NEAT1 led to cell cycle arrest in the G0/G1 phase. NEAT1 *via* miR-193b-3p/CCND1 axis could enhance the radio-resistance of cervical cancer.	(69)
Renal Cell Carcinoma (RCC)	NEAT1	102 pairs of CC and ANTs	miR-34a, c-Met, Caspase-3, Vimentin,N-cadherin,E-cadherin	–	Knockdown of NEAT1 led to a significant accumulation of cells at G1-phase. NEAT1 *via* the miR-34a/c-Met axis could enhance EMT and chemoresistance in RCC	(70)
Nasopharyngeal Carcinoma (NPC)	ROR	–	PCNA, Cyclin A, ZEB1/2, Vimentin,N-cadherin,E-cadherin, p53, p21	–	Knockdown of ROR could decrease the S and G2 phase population and increase the G1 phase population. ROR could promote proliferation, migration, and chemoresistance of NPC.	(71)
Ovarian Cancer (OC)	HOTAIR	Mouse/human; 11 pairs of OC and ANTs	Cyclin D1, CDK4	Wnt/β-catenin	Knockdown of HOTAIR could arrest the cell cycle at the G1 phase. Overexpression of HOTAIR by activating the Wnt/β-catenin pathway could lead to chemoresistance in human OC.	(71)
Pancreatic Adenocarcinoma (PA)	SNHG8	11 pairs of PA and ANTs	Caspase-3, PARP	–	Knockdown of SNHG8 could decrease the proliferative ability and prolonged G0/G1 phase in Hs766T and PANC-1 cells. Hence, SNHG8 could enhance the development and chemo-resistance of PA.	(72)
Breast Cancer	LINP1	67 pairs of breast cancer and ANTs	Cyclin D1, Cyclin D3, CDK4, p53, E-cadherin, Bax,N-cadherin, Vimentin, Caspase-8/9	–	Knockdown of LINP1 by inducing G1-phase cell cycle arrest and apoptosis mitigated breast cancer cell growth. Hence, LINP1 could act as an oncogene and promote chemoresistance in breast cancer.	(72)
Osteosarcoma (OS)	HOTTIP	21 pairs of OS and ANTs	Cyclin D1, CDK4	Wnt/β-catenin	Knockdown of HOTTIP by blocking the Wnt/β-catenin pathway could inhibit cell proliferation and arrest cell cycle at the G1 phase.	(73)

The importance of cell cycle-associated lncRNAs as diagnostic/prognostic markers have been assessed in several studies. Higher expression of NR2F2-AS1, PCAT6, FOXD-AS1, SNHG3, FLVCR-AS1, and some other lncRNAs has been associated with lower OS rate. **Table 3** summarizes the results of these studies.

**Table 3:** Role of cell cycle-associated lncRNAs as prognostic markers.

Sample Number	Kaplan-Meier Analysis	Univariate/Multivariate Cox Regression	Ref
60 pairs of PCa and ANTs	Higher expression of NR2F2-AS1 was associated with lower OS rate.	_	(37)
TCGA database	Higher expression of PCAT6 was associated with lower OS and DFS rates.	Higher expression of PCAT6 was associated with TNM stage.	(40)
105 pairs of HCC and ANTs	Higher expression of FOXD2-AS1 was associated with lower OS rate.	Higher expression of FOXD2-AS1 was associated with tumor number and tumor size.	(39)
20 pairs of HCC and ANTs	Higher expression of HOXD-AS1 was associated with lower OS rate.	Higher expression of HOXD-AS1 was associated with histologic grade.	(35)
77 ACC tissues and 30 normal	_	HOTAIR was an independent prognostic factor for DFS and OS of ACC patients.	(42)
60 pairs of glioma and ANTs	Higher expression of SNHG3 was associated with lower OS rate.	High expression of SNHG3 is an independent prognostic factor for glioma. Higher expression of SNHG3 was associated with KPS and tumor grade.	(33)
48 pairs of OS and ANTs	Lower expression of FLVCR-AS1 was associated with lower OS rate.	Lower expression of FLVCR-AS1 was associated with distant metastasis and size of tumor.	(47)
30 pairs of OS and ANTs	Higher expression of LINC01296 was associated with lower OS rate.	_	(48)
48 pairs of breast cancer and ANTs	Higher expression of RUSC1-AS1 was associated with lower OS rate.	Higher expression of RUSC1-AS1 was associated with TNM stage, tumor size, and lymphatic metastasis.	(51)
42 pairs of PaC and ANTs	Higher expression of TUG1 was associated with lower OS rate.	Higher expression of TUG1 was associated with TNM stage, tumor size, and lymphatic metastasis.	(53)
30 pairs of OSCC and ANTs	Higher expression of NEAT1 was associated with lower OS rate.	Higher expression of NEAT1 was associated with TNM stage, lymph node metastasis, and clinical stage.	(54)
75 pairs of CRC and ANTs	Higher expression of XIAP-AS1 was associated with lower OS rate.	Higher expression of XIAP-AS1 was associated with TNM stage.	(53)
60 pairs of CRC and ANTs	Higher expression of NR2F2-AS1 was associated with lower OS rate.	Higher expression of NR2F2-AS1 was associated with TNM stage and lymph node metastasis.	(56)
63 pairs of CRC and ANTs	Higher expression of NR2F2-AS1 was associated with lower OS rate.	_	
TCGA database	Higher expression of STEAP3-AS1 was associated with lower OS rate.	_	(59)
80 pairs of GC and ANTs	_	Higher expression of CASC11 was associated with TNM stage and lymph node metastasis.	(63)
60 pairs of NSCLC and ANTs	Higher expression of HNF1A-AS1 was associated with lower OS rate.	Higher expression of HNF1A-AS1 was associated with TNM stage and lymph node metastasis.	(64)
Bone marrow blood samples from 80 AML patients and 24 healthy controls	Higher expression of HOTTIP was associated with lower OS rate.	_	(65)
60 pairs of LSCC and ANTs	Higher expression of CDKN2B-AS1 was associated with lower OS rate.	Higher expression of CDKN2B-AS1 was associated with advanced clinical stage and lymph node metastasis.	(66)
72 pairs of CC and ANTs	Higher expression of NEAT1 was associated with lower OS rate.	Higher expression of NEAT1 was associated with TNM stage and lymph node metastasis.	(69)
102 pairs of CC and ANTs	Higher expression of NEAT1 was associated with lower OS and PFS rates.	Higher expression of NEAT1 was associated with TNM stage, tumor size, and lymph node metastasis.	(70)
11 pairs of PA and ANTs	Higher expression of SNHG8 was associated with lower OS rate.	Higher expression of SNHG8 was associated with tumor differentiation and clinical stage.	(72)
67 pairs of breast cancer and ANTs	Higher expression of LINP1 was associated with lower OS and DSF rates.	_	(72)

### miRNAs and Cell Cycle Control

These small transcripts participate in the regulation of cell cycle control *via* modulation of checkpoints and DNA repair mechanisms (16, 74). Moreover, they regulate expression of cyclins, CDKs, cyclin-dependent kinase inhibitors, and TF-associated proteins such as Rb (16). For instance, the miR-15a-16-1 cluster has been shown to induce cell cycle arrest at the G1 through suppressing expression of CDK1, CDK2, and CDK6 as well as D1, D3, and E1 cyclins (75–77). miR-188 suppress cell cycle transition at G1/S through inhibition of expression of cyclins D1, D3, E1, and A2 as well as CDK4 and CDK2. This miRNA also reduces Rb phosphorylation and decreases E2F transcriptional activity (78). miR‑424 has been shown to regulate cell cycle progression in the G2/M phase through inhibition of expression of CDK1 probably *via* the Hippo and the extracellular signal‑regulated kinase pathways (79). Moreover, regulation of CDK5 by miRNA-26a has been shown to control cell proliferation, apoptosis and tumor growth in an animal model of diffuse large B-cell lymphoma (80). Expression of CDK5 is also regulated by the tumor suppressor miRNA-505-5p in cervical cancer cells (81). Notably, this CDK has a distinct feature from other CDKs which is that it is not activated *via* interaction with cyclins. Instead, it is activated through binding with p35 and p39, or their cleaved proteins namely p25 and p29 (82).

Expression of cell cycle-related TFs is also regulated by miRNAs. For example, miR-17-92 cluster, miR-17-5p, miR-20a, miR-149*, miR-330, and miR-331-3p have been shown to suppress expression of E2F1, thus inducing cell cycle arrest in different tissues (83–86). On the other hand, miR-106a, miR-290, and miR-17-92 have been shown to target pRB or other proteins from this family (87–90).

**Figure 2:**
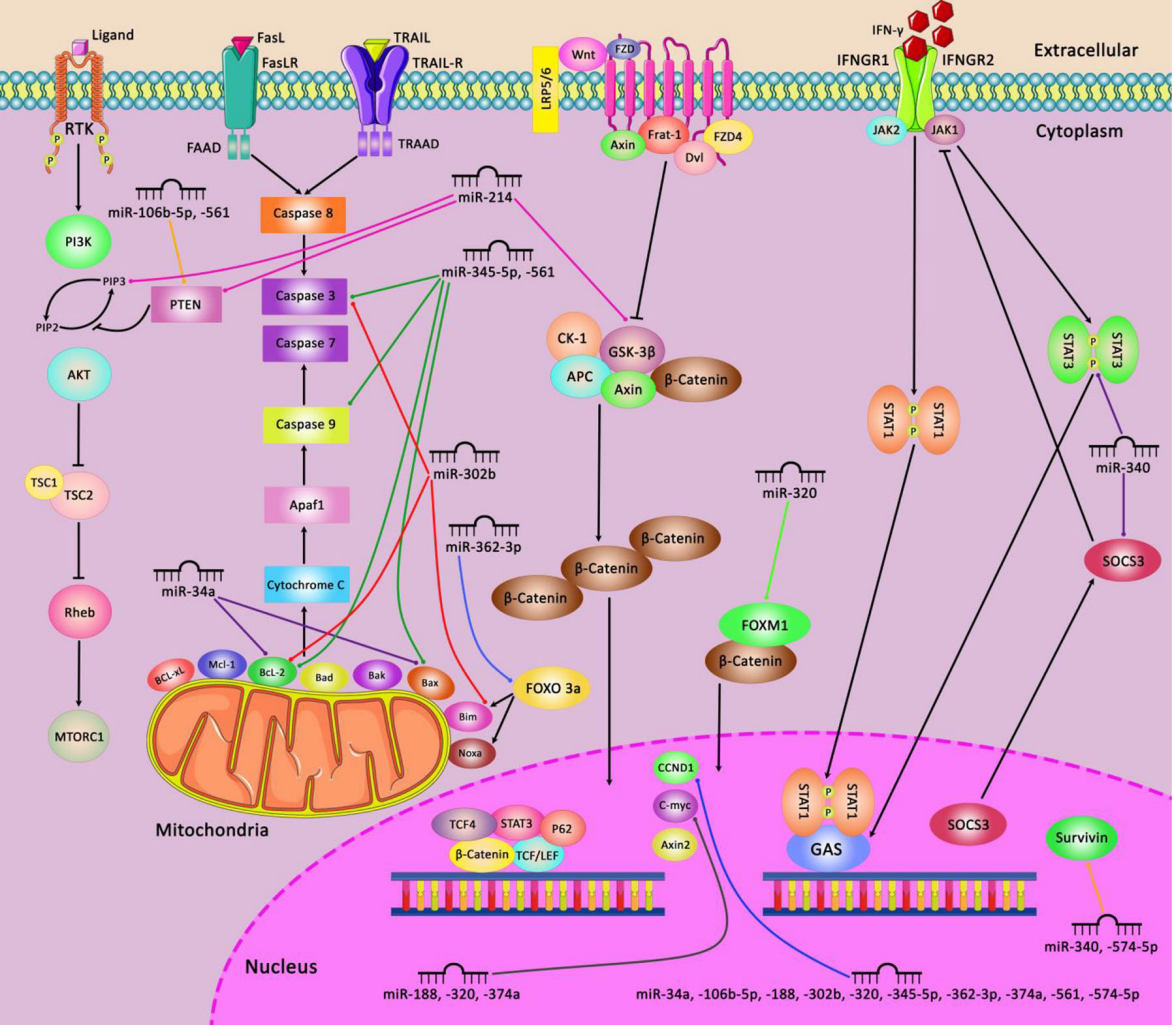
A schematic diagram of the regulation of mitochondrial apoptosis, Wnt/β-catenin, JAK-STAT, and PI3K/AKT signaling pathways *via* different miRNAs in various human cancers. Ectopic expression of some miRNAs including miR-345-5p, miR-561, miR-302b, miR-362-3p, and miR-34a could impede the mitochondrial apoptotic pathway *via* targeting caspase 3 and 9, Bcl-2, Bax, and Bim which can play an effective role in cell death suppression in variety of tumor cells (94, 95). Besides, miR-214, miR-320, miR-188, miR-374a, and miR-574-5p could activate the Wnt/β-catenin pathway in tumor cells through modulating GSK-3β, FOXM1, CCND1, and C-myc, and thereby promoting cell differentiation and proliferation as well as enhancing EMT and cell migration and invasion in different human cancers (96, 97). Additionally, miR-340, and miR-574-5p could regulate the JAK-STAT signaling pathway *via* targeting STAT3, SOCS3, and Survivin which have a significant role in regulating tumor cell growth and metastasis in various tumor cells (98, 99). In addition, aberrant expression of miR-214, miR-106b-5p, and miR-561 could negatively modulate PTEN and PIP3 in PI3K/AKT signaling pathway in different human cancers such as ovarian cancer, melanoma, and NSCLC cells (96, 100, 101).

A number of miRNAs such as miR-504 and miR-1285 regulate expression of p53 (91). Meanwhile, p53 has been shown to alter expression of several miRNAs such as miR-34a/b/c (92, 93). Thus, several miRNAs are implicated in the regulation of cell cycle progression through p53-mediated pathways. **Figure 2** illustrates the role of a number of miRNAs in the cell cycle control.

**Table 4** summarizes the function of miRNAs in cell cycle control.

**Table 4:** Function of miRNAs in cell cycle transition (ANTs, Adjacent normal tissues).

Cancer type	microRNA	Species and number of samples	Targets/Regulators/Signaling pathways	Function	Ref
Hepatocellular Carcinoma (HCC)	miR-23a-3p	mouse/human; 30 pairs of HCC and ANTs	PCDH17	MiR-23a-3p by targeting PCDH17 could promote G1/S cell cycle transition, cell growth, and metastasis and regulate chemosensitivity.	(102)
HCC	miR−214−3p	98 pairs of HCC and ANTs	MELK	MiR-214-3p by targeting MELK could decrease cell proliferation, induce cell cycle arrest at G1 phase, and enhance cell apoptosis.	(103)
HCC	miR-30b-5p	90 pairs of HCC and ANTs	DNMT3A, USP37, CCND1	MiR-30b-5p by targeting DNMT3A could repress proliferation, meanwhile by suppressing USP37 could decelerate cell cycle at G1 phase.	(104)
HCC	miR-3613-3p	GEO database	BIRC5, CDK1, NUF2, ZWINT, SPC24	MiR-3613-3p could affect cell proliferation and regulate cell cycle at G2/M phase.	(104)
Colon Cancer	miR-195-5p	42 pairs of colon cancer and ANTs	CDK8	MiR-195-5p by targeting CDK8 could inhibit cellular growth, suppress cellular migration and invasion, and induce cell cycle arrest at G1 phase.	(105)
Colon Cancer	miR-6734	_	p21	MiR-6734 by increasing p21 gene expression could induce cell cycle arrest and apoptosis in colon cancer cells.	(106)
Colorectal Cancer (CRC)	miR-4711-5p	mouse	KLF5, MDM2, TFDP1	MiR-4711-5p by targeting KLF5, MDM2, and TFDP1 could provoke G1 arrest, induce apoptosis, and suppress cell proliferation, migration and invasion, as well as stemness.	(107)
CRC	miR-193a-5p/-146 a-5p	_	MDM2, p53	MiR-193a-5p and miR-146 a-5p by targeting MDM2 could induce G1 arrest in CRC cells *via* p53.	(108)
CRC	miR‐744	mouse/human; 64 pairs of CRC and ANTs/TCGA dataset	RFC2, CCNE2	MiR‐744 by targeting RFC2/CCNE2 could inhibit proliferation and enhance G1/S arrest.	(109)
CRC	miR-133a-3p	20 pairs of CRC an ANTs	SENP1	MiR-133a-3p by targeting SNEP1 could inhibit cell proliferation and promote G1/S arrest.	(110)
CRC	miR−598	8 CRC and ANTs	INPP5E, CCND1, p27	MiR−598 by suppressing INPP5E could promote cell proliferation G1/S transition.	(111)
CRC	miR-1258	mouse/human; 60 pairs of CRC and ANTs	E2F8, CCND1, p21, p27, CDK2	MiR-1258 by directly targeting E2F8 could inhibit proliferation and enhance G0/G1 arrest.	(111)
Medulloblastoma	miR-221-3p	_	EIF5A2, CDK4, CCND1, Bcl-2, Bad	MiR-221-3p by targeting EIF5A2 could inhibit proliferation and promote G0/G1 arrest and apoptosis.	(112)
Glioblastoma multiforme (GBM)	miR-1179	mouse/human; 89 GBM tissues and 10 normal brain tissues/CGGA database	E2F5, CDK2, CDK6	MiR-1179 by targeting E2F5 could inhibit glioblastoma cell proliferation and cell induce G0/G1 arrest.	(113)
glioma	miR-1468-5p	mouse/CGGA database	RRM1, AKT/ERK	MiR-1468-5p by targeting RRM1 could inhibit glioma cell proliferation and induce G1/S arrest.	(113)
glioma	miR-520d-5p	mouse/human; 31 glioma tissues and 8 normal brain tissues/CGGA and TCGA databases	PTTG1	MiR-520d-5p by targeting PTTG1 could inhibit human glioma cell proliferation and induce G0/G1 arrest.	(114)
glioma	miR-519d-3p	20 pairs of glioma and ANTs	CCND1	MiR-519d-3p by targeting CCND1 could inhibit cell proliferation and cell cycle G1/S transition.	(107)
glioma	miR-940	mouse/human; 14 low grade glioma tissues, 18 GBMs and 7 non-cancerous brain tissues/CGGA database	CKS1, CDC2, CDK2, CyclinE1	MiR-940 by targeting CKS1could inhibit glioma cells proliferation and G0/G1 phase transition.	(115)
glioma	miR−770	63 pairs of glioma and ANTs	CDK8, Wnt/β-catenin	MiR-770 by targeting CDK8 could inhibit glioma cell proliferation and cell cycle G1/S transition and induce apoptosis *via* suppressing Wnt/β-catenin signaling.	(111)
glioma	miR-129-5p	17 glioma tissues and 9 normal brain tissues	DNMT3A, Cyclin A2, CDK2	MiR-129-5p by directly targeting DNMT3A could inhibit cell proliferation and induce G1 phase arrest in glioma.	(110)
glioma	miR-188	81 glioma tissues and 26 normal brain tissues	c-Myc, Cyclin D1, β-catenin	MiR-188 by targeting β-catenin could inhibit proliferation and G1/S transition.	(110)
Gastric Cancer (GC)	miR-383	60 pairs of GC and ANTs	CCNE2	MiR-383 by targeting Cyclin E2 could inhibit proliferation and enhance G1 arrest and apoptosis.	(116)
GC	miR-129-5p	mouse/human; 60 pairs of GC and ANTs/TCGA dataset	HOXC10, Cyclin D1	MiR-129-5p by targeting HOXC10/Cyclin D1could suppress GC cell proliferation and facilitate G1/S cell cycle transition.	(117)
GC	miR-340	42 pairs of GC and ANTs	SOCS3, p-STAT3, Survivin, JAK-STAT	MiR-340 by targeting SOCS3 could enhance cell proliferation, G1/S transition, and attenuate apoptosis in GC cells *via* JAK-STAT signaling pathway.	(99)
GC	miR-218	mouse/126 pairs of GC and ANTs	CDK6, CyclinD1, E2F1, SLIT2, SLIT3	MiR-218 could suppress gastric cancer cell cycle progression at G1 phase through CDK6/Cyclin D1/E2F1 axis in a feedback loop.	(114)
Pancreatic Cancer (PC)	miR-144-3p	40 pairs of PC and ANTs	PRR11, p-JNK, p-p38, p-ERK, CyclinD1, Cdc25A, p21 MAPK	MiR-144-3p by targeting PRR11 could induce cell cycle arrest and apoptosis in pancreatic cancer cells *via* MAPK signaling pathway.	(118)
Pancreatic Ductal Carcinoma	miR-590-3p	60 pairs of pancreatic ductal carcinoma and ANTs	PPP2R2A,	MiR-590-3p by directly inhibiting p27 and PPP2R2A could promote the development of pancreatic cancer *via* the G1/S cell cycle pathway.	(119)
Pancreatic Adenocarcinoma (PDAC)	miR-17-5p	mouse/human; 26 pairs of PDAC and ANTs	RBL2, E2F4	MiR-17-5p by targeting RBL2 could enhance PDAC proliferation by accelerating G1/S phase transition *via* disruption of RBL2/E2F4-repressing complexes.	(120)
Lung Cancer	miR‐377‐5p	30 pairs of lung cancer and ANTs	AKT1, CCND1, fibronectin, vimentin, Foxo1, p27kip1, p21Cip1, E‐cadherin	MiR‐377‐5p by targeting AKT1 could inhibit proliferation, invasion, and G1/S phase transition *via* suppressing EMT.	(121)
Lung Adenocarcinoma (LUAD)	miR-486-5p	mouse/human; 76 pairs of LUAD and ANTs/GEO and TCGA databases	NEK2 E-cadherin, N-cadherin, vimentin, MMP-2, MMP-9	MiR-486-5p by targeting NEK2 could attenuate proliferation, and confer G0/G1 arrest and also inhibit metastasis *via* suppressing EMT.	(122)
Non-Small Cell Lung Cancer (NSCLC)	miR-561	68 pairs of NSCLC and ANTs	P-REX2a, PTEN, Cyclin D1, CDK2, Bcl-2, Bax, caspase 9/3, AKT	MiR-561 by targeting P-REX2a could suppress cell proliferation, induce G1/S arrest and apoptosis *via* the PTEN/AKT signaling.	(100)
NSCLC	miR-7-5p	85 pairs of NSCLC and ANTs	PAK2	MiR-7-5p by targeting PAK2 could induces cell growth inhibition, G0/G1 phase arrest, and apoptosis.	(123)
NSCLC	miR‐34b‐3p	100 pairs of NSCLC and ANTs/GEO database	CDK4	MiR‐34b‐3p by targeting CDK4 could repress cell proliferation, G1 phase transition, and cell apoptosis.	(123)
NSCLC	miR-613	mouse/human; 56 pairs of NSCLC and ANTs	CDK4	MiR-613 by targeting CDK4 could induce cell cycle arrest in the G1/S phase.	(124)
Osteosarcoma (OS)	miR−106b−5p	18 pairs of OS and ANTs	CDKN1A, p21	MiR−106b−5p by targeting CDKN1A could promote cell proliferation and G0/G1 transition in OS.	(117)
OS	miR-671-5p	mouse/human; 20 pairs of OS and ANTs	CCND1, CDC34	MiR-671-5p by targeting CCND1 and CDC34 could inhibit proliferation and G1/S transition.	(125)
OS	miR-299-5p	_	Cyclin D, Cyclin E, CDK, P16, P21	MiR-299-5p *via* targeting cyclin E, cyclin D, CDK could mediate G1 phase, and promote cell proliferation and cancer progression.	(126)
OS	miR-34a	_	DUSP1, Bax, Bcl-2, CCNE, CCND1, E-cadheri, β-catenin	MiR-34a by targeting DUSP1 could decrease proliferation, adhesion, cell cycle arrest in G0/G1 phase and cell apoptosis.	(94)
OS	miR-22	_	_	MiR-22 could induce G0/G1 cellular cycle arrest, thus leading to apoptosis of OS cells.	(127)
cervical cancer (CC)	miR-140-3p	mouse/human; 44 pairs of CC and ANTs/TCGA database	RRM2, Cyclin A, Cyclin B1, Cyclin D1, PI3K	MiR-140-3p by targeting RRM2 could impede the proliferation of human cervical cancer cells to induce cell-cycle arrest and early apoptosis.	(128)
CC	miR-92a	74 pairs of CC and ANTs	p21	MiR-92a by inhibiting p21 could promote cell proliferation and G1/S transition.	(129)
Epithelial Ovarian Cancer (EOC)	miR-337-3p	mouse/human; 105 pairs of EOC and ANTs	PIK3CA, PIK3CB, PI3K/Akt pathway	MiR-337-3p by targeting PIK3CA and PIK3CB could inhibit proliferation and induce apoptosis and cell cycle arrest in G0/G1 phase.	(122)
ovarian cancer (OC)	miR-107	mouse	CCNE1	MiR-107 by directly targeting cyclin E1could induce G1/S phase arrest.	(130)
OC	miR−214	124 pairs of OC and ANTs	PTEN, PIP3, GSK−3β, PI3K/Akt pathway	MiR−214 by targeting PTEN could promote cell proliferation and G1/S arrest and inhibit apoptosis *via* PI3K/Akt signaling pathway.	(96)
Renal Cell Carcinoma (RCC)	miR-362-3p	mouse/human; 25 pairs of RCC and ANTs	SP1, FOXO3, p-RB, CCND1, Snail, AKT	MiR-362-3p by targeting SP1 could enhance G1 arrest and inhibit EMT Progression in RCC *via* Akt/FOXO3 signaling.	(122)
clear cell Renal Cell Carcinoma (ccRCC)	miR-181a	42 pairs of ccRCC and ANTs	KLF6	MiR-181a by targeting KLF6 expression could enhance cell proliferation, accelerate G1/S transition, and diminish apoptosis.	(131)
Breast Cancer (BC)	miR‐99a‐5p	mouse/human; 84 pairs of BC and ANTs	CDC25A, marker ki67, Cyclin D1, p21	MiR‐99a‐5p by downregulating CDC25A could suppress breast cancer progression and G1/S transition.	(132)
BC	miR-1301-3p	60 pairs of BC and ANTs	ICT1, Bad, Bax, Bcl-2, CDK4, CCND1	MiR-1301-3p by targeting ICT1 could inhibit cell proliferation and promote G0/G1 arrest and apoptosis.	(133)
BC	miR-543	_	ERK2, RSK2, MSK1, ERK/MAPK	MiR-543 by targeting ERK2 could suppress breast cancer cell proliferation, block cell cycle, and induce cell apoptosis *via* regulating ERK/MAPK pathway.	(134)
Prostate Cancer (PCa)	miR-501-3p	22 pairs of PCa and ANTs	CREPT, CCND1	MiR-501-3p by targeting CREPT/CCND1 could restrict prostate cancer growth and increase G0/G1 arrest.	(135)
PCa	miR−26a, miR−138	_	EZH2, CCNE2, CCND1, CCND3, CDK6	MiR−26a and miR−138 by regulating EZH2 could block the G1/S transition by targeting the cell cycle network.	(136)
Acute Lymphoblastic Leukemia (ALL)	miR-144	mouse/human; 59 ALL PB samples and 47 normal control samples	FMN2	MiR-144 by targeting FMN2 could regulate cancer cell proliferation and G1/S transition.	(137)
Acute Myeloid Leukemia (AML)	miR−192	mouse/human; 10 AML BM tissues	CCNT2, p16, p21, p27	MiR−192 by targeting CCNT2 could induce G0/G1 arrest, cell differentiation, and apoptosis.	(138)
AML	miR‐345‐5p	29 AML PB samples, and 29 healthy PB samples	AKT2, CCND1, CDK4, CDK6, Rb, Bax, Bcl-2, PARP, caspase3, PI3K/Akt	MiR‐345‐5p by targeting AKT2 could decrease proliferation and enhance G0/G1 arrest and apoptosis.	(135)
_	miR-4779	mouse/human; 10 pairs of colon cancer and ANTs	PAK2, CCND3	MiR-4779 by targeting PAK2 and CCND3 could suppress tumor growth by inducing apoptosis and G1/S arrest.	(139)
_	miR-5582-5p	mouse	GAB1, SHC1, CDK2	MiR-5582-5p by targeting GAB1, SHC1, and CDK2 could induce apoptosis and cell cycle arrest at G1 phase in cancer cells.	(140)
Head and Neck Squamous Cell Carcinoma (HNSCC)	miR-34a	39 pairs of HNCC and ANTs	FUT1, AXL, MAP2K1, AREG, p53, MAPK, ErbB	MiR-34a could suppress HNSCC growth, induce G0/G1 arrest and promote senescence *via* MAPK, ErbB, and p53 signaling pathways.	(141)
Oral Squamous Cell Carcinoma (OSCC)	miR-155	46 OSCC tissues and 25 normal control	p27Kip1	MiR-155 by regulating p27Kip1 could promote proliferation and inhibit G1 arrest and apoptosis.	(142)
OSCC	miR-376c-3p	49 pairs of OSCC and ANTs	HOXB7	MiR-376c-3p by suppressing HOXB7 could inhibit proliferation, invasion, migration, cell cycle at G0/G1 phase, and apoptosis of OSCC cells.	(143)
Nasopharyngeal Carcinoma (NPC)	miR-130a-3p	mouse/human; 56 NPC tissues and 45 normal nasopharyngeal tissues	BACH2, E-cadherin, Vimentin, N-cadherin	MiR-130a-3p by suppressing BACH2 could inhibit the viability, proliferation, invasion, and cell cycle in G0/G1 phase, and promote apoptosis of NPC cells *via* regulating EMT pathway.	(141)
NPC	miR−150	mouse/human; 8 NPC and ANTs	CCND1, CCND2, CDK2, CCNE2	MiR−150 by directly targeting CCND1, CCND2, CDK2, and CCNE2 could inhibit proliferation and tumorigenicity *via* retarding G1/S phase transition in NPC.	(144)
Esophageal Squamous Cell Carcinoma	miR-219-5p	20 pairs of ESCC and ANTs	CCNA2	MiR-219-5p by targeting CCNA2 could suppress cell proliferation and induce G2/M arrest.	(145)
Esophageal Carcinoma (EC)	miR-29c-3p	30 pairs of EC and ANTs	CCNA2, p53	MiR-29c-3p by targeting CCNA2 could promote G1/S arrest, migration and invasion in EC cells *via* p53 signaling.	(146)
Papillary Thyroid Cancer	miR-1256	49 pairs of PTC and ANTs	HTR3A	MiR-1256 by directly targeting HTR3A could suppress cell proliferation and induce cell cycle G0/G1 phase arrest.	(147)
Thyroid Cancer	miR−574-5p	_	pan−QKI, QKI5/6/7, cyclin D1, Survivin, Wnt/β-catenin	MiR−574-5p by targeting QKI proteins could promote G1/S transition and apoptosis in thyroid cancer cells *via* Wnt/β-catenin signaling pathway.	(99)
Thyroid cancer	miR-144	mouse/human; 64 pairs of thyroid cancer and ANTs/TCGA database	E2F8, CCND1	MiR-144 by targeting E2F8 could inhibit thyroid cancer progression *via* retarding G1/S transition.	(148)
Thyroid Cancer	miR-639	59 pairs of thyroid cancer and ANTs	CDKN1A	MiR-639 by suppressing CDKN1A could promote cell proliferation and cell cycle.	(149)
melanoma	miR−106b−5p	18 melanoma tissues and 18 benign nevi tissues	PTEN, p27Kip1, Cyclin D1, Akt/ERK	MiR−106b−5p by targeting PTEN could accelerate G1/S transition and promote cell cycle progression of malignant melanoma *via* Akt/ERK signaling pathway.	(101)
uveal melanoma (UM)	miR-181b	3 UM and 3 normal uvea tissues	CTDSPL, Rb, E2F1	MiR-181b by targeting CTDSPL could promote cell cycle and G1/S transition *via* increasing pRB/E2F1.	(111)
Retinoblastoma (RB)	miR-133a-3p	mouse/human; 60 pairs of RB and ANTs	CREB1, Cyclin B1, Cyclin D1, caspase3	MiR-133a-3p by targeting CREB1 could promote apoptosis and induce G0/G1 arrest.	(150)

The interaction between miRNAs and cell cycle controlling proteins has implications in defining response to chemotherapeutic agents. For instance miR-192/miR-215 and miR-320 could affect response of cancer cells to 5-Fluorouracil resistance (151, 152). In addition, miR-100 could increase cisplatin sensitivity, inhibit cell proliferation, induce conversion from G1 to S phase and promote apoptosis through directly targeting mTOR and PLK1 (140). miR-374a is another miRNA which alters chemoresistance phenotype in nasopharyngeal carcinoma. In this type of cancer, miR-374a decreases proliferation, migratory aptitude, invasiveness, metastatic ability, and resistance to cisplatin. Functionally, miR-374a decreases expression of CCND1 to attenuate activity of the pPI3K/pAKT/c-JUN axis through making a negative-feedback circle. This miRNA also inhibits downstream signals associated with cell cycle transition (144). miR-9600 has been demonstrated to attenuate tumorigenesis and metastatic potential of lung cancer cells *via* inhibiting expression of STAT3. Besides, miR-9600 improved response of cancer cells to paclitaxel and cisplatin through this axis and enhancement of chemotherapy-associated apoptosis (153). Besides, miR-106b-5p has a crucial impact in modulation of cisplatin resistance in lung cancer through inhibiting expression of PKD2 (154). **Table 5** summarizes the data regarding the role of miRNAs in conferring resistance to chemotherapeutic agents.

**Table 5:** Role of cell cycle regulating miRNAs in conferring resistance to chemotherapeutic agents.

Cancer type	microRNA	Human/Animal	Targets/Regulators	Involved pathways	Function	Ref
HCC	miR-122	_	MDR1, Bcl-w, CCNG1, Cyclin B1	_	MiR-122 with chemotherapeutic agents by targeting MDR related genes and Cyclin B1 could inhibit HCC cell growth *via* inducing G2/M arrest.	(155)
HCC	miR-101	mouse/human; 93 pairs of HCC and ANTs	EZH2	_	MiR-10 by targeting EZH2 could inhibit HCC progression, induce cell cycle arrest at G1 phase and increase drug-induced apoptosis.	(156)
CRC	miR-192/miR-215	_	TYMS, p53, p27, p21, CDK6, CDK2, CCND1, CCNE,	_	MiR-192/miR-215 by targeting TYMS could influence 5-Fluorouracil resistance and reduce cell proliferation and promote G0/G1 arrest in CRC cells.	(152)
colon cancer	miR-195	_	CHK1, WEE1, CCNB1, GFP	_	MiR-195 by targeting CHK1, and WEE1could promote acquisition of drug resistance to 5-FU and accelerate G2/M transition in colon cancer cells.	(157)
colon cancer	miR-320	50 pairs of colon cancer and ANTs	FOXM1, Cyclin D1, c-MYC	Wnt/β-catenin	MiR-320 by targeting FOXM1 could enhance the sensitivity of human colon cancer cell to 5-Fu and Oxaliplatin and induce cell cycle arrest at G0/G1 phase.	(151)
NPC	miR-374a	mouse/human; 70 fresh NPC tissues, 20 fresh nasopharynx tissues and 149 paraffin-embedded NPC tissues	CCND1, c-MYC, c-JUN, PDCD4, E-cadherin, N-cadherin, Snail, E2F1, Rb	PI3K/Akt, β-catenin	MiR-374a by targeting CCND1could retard G1/S transition and suppress cell growth, metastasis, and sensitize NPC cells to cisplatin *via* PI3K/Akt signaling pathway.	(144)
NPC	miR-16	mouse/human; 63 fresh NPC and 15 NP tissues	CDK4, c-Myc, E2F1	_	MiR-16a by targeting CCND1 could inhibit cell cycle progression at G1/S phase and sensitize NPC cells to chemotherapy.	(158)
GC	miR-647	16 pairs of GC and ANTs	ANK2, FAK, MMP2, MMP12, CD44, SNAIL1	_	MiR−647 by targeting ANK2 could ameliorate drug resistance and metastasis and promotes cell cycle arrest at the G0/G1 phase in GC cells.	(159)
GC	miR-31	_	ZH2, E-cadherin, N-cadherin, vimentin	_	MiR-31 by targeting ZH2 could trigger G2/M cell cycle arrest, enhance the chemosensitivity and inhibit migration and invasion of human gastric cancer cells.	(160)
Malignant Mesothelioma (MM)	miR-34a	_	_	_	MiR-34a could promote cell cycle arrest at G2 and cell death in the presence of docetaxel in mesothelioma.	(161)
Epithelial Ovarian Cancer (EOC)	miR-100	mouse	PLK1	mTOR	MiR-100 by directly targeting mTOR and PLK1 could increase cisplatin sensitivity, inhibit cell proliferation, induce conversion from G1 to S phase, and promote apoptosis.	(140)
PC	miR-373-3p	GEO database	CCND2, GADD45A, CDC6, CCNB1, p21, p53	_	MiR-373-3p by targeting CCND2 could inhibit cell propagation, migration, and invasion, and boost apoptosis, chemosensitivity, and G0/G1 arrest in gemcitabine resistance pancreatic carcinoma cells.	(162)
Pancreatic Adenocarcinoma	miR-21/-221	_	PTEN, RECK, p27kip1	_	Antisense inhibition of miR-21/-221 could arrest cell cycle at G1 phase and induce apoptosis, and sensitize the effects of Gemcitabine in pancreatic adenocarcinoma.	(163)
BC	miR-26a, miR-30b	_	CCNE2	_	Trastuzumab could upregulate miR-26a and miR-30b in BC. MiR-26a and miR-30b by targeting CCNE2 could induce cell growth suppression and G1 arrest.	(164)
BC	miR-302b	_	E2F1, ATM, PARP, Caspase 3	_	MiR-302b by regulating E2F1/ATM axis could enhance breast cancer cell sensitivity to cisplatin and promote G1/S arrest in BC cells.	(165)
_	miR-16, miR-26a	public databases	Wee1, Chk1, Cyclin E	_	MiR-16 and miR-26a by targeting Wee1 and Chk1 could retard G2/M arrest and by targeting Cyclin E could induce G1/S arrest in response to p53 activation by genotoxic stress.	(166)
melanoma	miR-30a-5p	_	IGF1R, p53	Akt	MiR-30a-5p by targeting IGF1R could confer cisplatin resistance and induce cell cycle arrest at G2/M phase by regulating Akt activity and protein level of p53.	(162)
NSCLC	miR-9600	20 fresh NSCLC tissues, 124 FFPE tissues	STAT3, RB, CDK2, Cylin D1, Cyclin E, Bcl2, Mcl-1, Bcl-xL, caspase-3, caspase-7	_	MiR-9600 by targeting STAT3 could suppress tumor progression, induce cell cycle arrest at G1/S phase, and promote paclitaxel sensitivity in NSCLC.	(153)
NSCLC	miR-106b-5p	40 NSCLC tissues (20 cisplatin resistant and 20 cisplatin sensitive)	PKD2	_	MiR-106b-5p by targeting PKD2 could reduce the resistance to cisplatin, increase cell apoptosis, and promote G1/S phase transition in NSCLC cells.	(154)
OS	miR-302b	_	caspase-3, Bcl-2, Bim, cyclin D1, CDK2, CDK4	Akt	Epirubicin-mediated expression of miR-302b by regulating cyclin D1 and CDK2/4 could arrest cell cycle at G1 phase and induce apoptosis by caspase-3 activation in OS cells.	(167)

Cell cycle-regulating miRNAs can be used as prognostic markers in cancer. Low expression of several miRNAs such as miR-129-5p, miR-29c-3p, miR-140-3p, miR-7-5p, miR-940, miR-107, miR-671-5p, and iR-299-5p has been associated with shorter survival rate of patients with certain types of cancers. **Table 6** summarizes the results of studies which assessed this aspect of miRNAs.

**Table 6:** The role of cell cycle controlling miRNAs as diagnostic/prognostic markers in cancer.

Sample number	Kaplan-Meier analysis	Multivariate cox regression	Ref
TCGA dataset of GC patients	Low expression of miR-129-5p was associated with shorter OS rate.	_	(117)
30 EC patients	Low expression of miR-29c-3p was associated with shorter OS rate.	_	(146)
CC patients from TCGA database	Low expression of miR-140-3p was associated with shorter OS rate.	_	(128)
85 NSCLC patients	Low expression of miR-7-5p was associated with shorter OS rate.	Low expression of miR-7-5p was correlated with advanced TNM stage.	(123)
glioma patients from CGGA database	Low expression of miR-940 was associated with shorter OS rate.	_	(115)
OC patients from TCGA database	Low expression of miR-107 was associated with shorter OS rate.	_	(130)
20 OS patients	Low expression of miR-671-5p was associated with shorter OS rate.	_	(125)
OS patients from GEO database	High expression of miR-299-5p was associated with shorter OS rate.	_	(126)
26 PDAC patients	High expression of miR-17-5p was associated with shorter OS rate.	_	(120)
glioma patients from CGGA database	Low expression of miR-1468-5p was associated with shorter OS rate.	_	(113)
GBM patients from CGGA database	Low expression of miR-1179 was associated with shorter OS rate.	_	(113)
GC patients from databases	Low expression of miR-218 was associated with shorter OS rate.	_	(114)
98 HCC patients	Low expression of miR-214-3p was associated with shorter OS and RFS rates.	_	(103)
90 HCC patients	Low expression of miR-30b-5p was associated with shorter OS rate.	_	(104)
124 NSCLC patients	Low expression of miR-9600 was associated with shorter OS rate.	Expression of miR-9600 was correlated with advanced TNM stages and lymph node involvement.	(153)

## Discussion

Several lncRNAs and miRNAs have been shown to regulate cell cycle at different stages thus influencing the proliferation rate. Abnormal function of these transcripts might lead to the development of human cancers. Cell cycle is regulated by several lncRNAs through epigenetic modulation of gene expression regulation of transcription factors modulation of translation mRNA stability, and enhancement of protein-protein interactions (15). Recruitment of EZH2 to the promoter region of target genes is a common mechanism of action of some of these lncRNAs. Dysregulation of cell cycle-related lncRNAs is regarded as a hallmark of cancer. A number of these lncRNAs also contribute in controlling the proliferation rate of normal differentiated cells or during organogenesis, therefore being important in the context of regenerative medicine and in cell senescence studies. For instance, Malat1 controls differentiation of myogenic cells and muscle regeneration (168). In addition, H19 regulates myoblast differentiation and muscle regeneration (169). However, most of the cell-cycle regulatory roles of lncRNAs have been assessed in the context of cancer.

miRNAs also exert functional roles in the regulation of cell cycle. A comprehensive genome-wide screen of cell cycle-associated miRNAs has led to identification of a distinctive group of miRNAs that target almost all cyclins/CDKs. These miRNAs are extremely potent in impeding cancer cell proliferation (170). Systemic administration of a number of these miRNAs using nanoparticle delivery methods has repressed tumor progression in a number of xenograft models indicating the role of these miRNAs in the treatment of cancer (170). The prominent role of cell cycle-regulatory miRNAs in the pathogenesis of cancer has been further highlighted by the observed dysregulation of these transcripts in the cancer stem cells (CSCs) (171). These miRNAs have been shown to target several genes that regulate cell cycle progression among them are *PTEN* (172), *JAK1*, *SOX4*, *STAT3*, *AKT*, *EZH1*, *HMGA2* (173), *CDK4/6*, *NOTCH1* (174), and *ZEB1/2* (175). Therefore, cell-cycle regulatory miRNAs represent important targets for intervention with the invasive and metastatic properties of cancer cells which are associated with CSC phenotypes. Furthermore, identification of miRNAs with distinctive functions in CSCs and normal stem cells would facilitate design of specific targeted therapies for cancer patients with fewer side effects in normal tissues. This research avenue needs to be explored in future studies.

As lncRNAs have important roles in the regulation of activity of miRNAs through serving as molecular sponges for these small transcripts, identification of this type of interactions between cell cycle-related lncRNAs and miRNAs would pave the way for better recognition of the molecular mechanism of cell cycle progression. Several lncRNAs act as competing endogenous RNAs for miRNAs, thus regulating expression of cell cycle-associated miRNAs. Examples include (but not limited to): loc285194/miR-211 (176), HOTAIR/miR-1 (23), HOTAIR/miR-206 (24), and ANRIL/miR-384 (177). In addition to this kind of interaction between lncRNAs and miRNAs, some lncRNAs serve as precursors for miRNAs. Such situation exists between H19 and miR-675 (178). While E2F1 enhances expression of H19 lncRNA (179), miR-675 suppresses pRB expression (180). In turn, pRB inhibits E2F-associated transcription of H19 constructing a self-regulated network between H19 and pRB (13). These examples obviously show the complex network between lncRNAs, miRNAs, and TFs.

Expression of cell cycle-associated non-coding RNAs directly influences the survival of patients with diverse cancer types. This speculation is based on the obtained data from both high and low throughput studies. An example of former type of studies is a study which assessed RNA seq data of a large cohort of patients with colorectal cancer. Authors have reported several differentially expressed cell cycle genes and miRNAs which regulate expression of these genes. Subsequently, they verified correlations between expression levels of these genes/miRNAs and patients’ survival (181). Moreover, these non-coding RNAs can contribute in the construction of diagnostic panels for diverse types of cancers.

Taken together, cell cycle-associated lncRNAs/miRNAs are potential therapeutic targets for management of cancer and possible biomarkers for prediction of cancer course. However, lack of specificity in the regulatory roles of some of these transcripts limits their application in the clinical settings. Future studies should focus on identification of the network between these two kinds of transcripts and TFs using high throughput techniques. The results of these studies would fulfill the prerequisite step for design of targeted therapies.

## Author Contributions

SG-F and MT wrote the manuscript and contributed in study design. HS and FTA collected the data and designed the tables. All authors contributed to the article and approved the submitted version.

## Conflict of Interest

The authors declare that the research was conducted in the absence of any commercial or financial relationships that could be construed as a potential conflict of interest.
